# Discovery of a Novel Antimicrobial Peptide from *Paenibacillus* sp. Na14 with Potent Activity Against Gram-Negative Bacteria and Genomic Insights into Its Biosynthetic Pathway

**DOI:** 10.3390/antibiotics14080805

**Published:** 2025-08-06

**Authors:** Nuttapon Songnaka, Adisorn Ratanaphan, Namfa Sermkaew, Somchai Sawatdee, Sucheewin Krobthong, Chanat Aonbangkhen, Yodying Yingchutrakul, Apichart Atipairin

**Affiliations:** 1 School of Pharmacy, Walailak University, Thasala, Nakhon Si Thammarat 80160, Thailand; nuttapon.so@wu.ac.th (N.S.); namfa.se@wu.ac.th (N.S.); somchai.sa@wu.ac.th (S.S.); 2 Drug and Cosmetics Excellence Center, Walailak University, Thasala, Nakhon Si Thammarat 80160, Thailand; 3 Department of Pharmaceutical Chemistry, Faculty of Pharmaceutical Sciences, Prince of Songkla University, Hat Yai, Songkhla 90112, Thailand; adisorn.r@psu.ac.th; 4 Center of Excellence in Natural Products Chemistry (CENP), Department of Chemistry, Faculty of Science, Chulalongkorn University, Bangkok 10330, Thailand; sucheewin.k@chula.ac.th (S.K.); chanat.a@chula.ac.th (C.A.); 5 Center of Excellence on Petrochemical and Materials Technology, Chulalongkorn University, Bangkok 10330, Thailand; 6 National Center for Genetic Engineering and Biotechnology, National Science and Technology Development Agency, Pathum Thani 12120, Thailand; yodying.yin@biotec.or.th

**Keywords:** antimicrobial peptides, biosynthetic gene clusters, non-ribosomal peptide synthetase, *Paenibacillus* sp. Na14, soil bacteria

## Abstract

Background/Objectives: Antimicrobial resistance (AMR) contributes to millions of deaths globally each year, creating an urgent need for new therapeutic agents. Antimicrobial peptides (AMPs) have emerged as promising candidates due to their potential to combat AMR pathogens. This study aimed to evaluate the antimicrobial activity of an AMP from a soil-derived bacterial isolate against Gram-negative bacteria. Method: Soil bacteria were isolated and screened for antimicrobial activity. The bioactive peptide was purified and determined its structure and antimicrobial efficacy. Genomic analysis was conducted to predict the biosynthetic gene clusters (BGCs) responsible for AMP production. Results: Genomic analysis identified the isolate as *Paenibacillus* sp. Na14, which exhibited low genomic similarity (61.0%) to other known *Paenibacillus* species, suggesting it may represent a novel species. The AMP from the Na14 strain exhibited heat stability up to 90 °C for 3 h and retained its activity across a broad pH range from 3 to 11. Structural analysis revealed that the Na14 peptide consisted of 14 amino acid residues, adopting an α-helical structure. This peptide exhibited bactericidal activity at concentrations of 2–4 µg/mL within 6–12 h, and its killing rate was concentration-dependent. The peptide was found to disrupt the bacterial membranes. The Na14 peptide shared 64.29% sequence similarity with brevibacillin 2V, an AMP from *Brevibacillus* sp., which also belongs to the Paenibacillaceae family. Genomic annotation identified BGCs associated with secondary metabolism, with a particular focus on non-ribosomal peptide synthetase (NRPS) gene clusters. Structural modeling of the predicted NRPS enzymes showed high similarity to known NRPS modules in *Brevibacillus* species. These genomic findings provide evidence supporting the similarity between the Na14 peptide and brevibacillin 2V. Conclusions: This study highlights the discovery of a novel AMP with potent activity against Gram-negative pathogens and provides new insight into conserved AMP biosynthetic enzymes within the Paenibacillaceae family.

## 1. Introduction

Infections caused by ESKAPE pathogens (*Enterococcus faecium*, *Staphylococcus aureus*, *Klebsiella pneumoniae*, *Acinetobacter baumannii*, *Pseudomonas aeruginosa*, and *Enterobacter* spp.) pose a significant global public health threat due to their high morbidity and mortality rates [1]. The World Health Organization (WHO) identifies them as critical concerns because they quickly develop resistance to many antibiotics. From 1990 to 2021, drug-resistant infections, including sepsis, have been linked to an estimated 15 to 20 million deaths worldwide [2]. The WHO has implemented action plans aimed at mitigating the impact of multidrug-resistant infections. These plans emphasize improving awareness of antimicrobial resistance, promoting the rational use of antimicrobial agents, and encouraging the discovery of novel antimicrobial compounds [3].

Antimicrobial peptides (AMPs) are essential natural compounds produced by living organisms to protect themselves against pathogenic infections [4]. These peptides are composed of amino acid sequences that typically combine both polar and non-polar residues, resulting in amphipathic properties that enable them to target and disrupt the membranes of pathogenic cells. AMPs isolated from natural sources are highly diverse, particularly among microbes, leading to a wide variety of AMP classes with distinct mechanisms of action. Their physical interaction with bacterial membranes, involving both polar and hydrophobic regions, allows AMPs to penetrate and destabilize membrane integrity through various models such as the carpet, toroidal pore, barrel-stave, and aggregate mechanisms [5]. The ability of AMPs to physically interact with cell membranes reduces the likelihood of pathogens developing resistance, especially when compared to traditional antibiotics that target specific receptor proteins. This characteristic makes AMPs a promising strategy for combating antibiotic-resistant infections [6].

Soil bacteria have emerged as valuable sources for antibiotic discovery. For example, polymyxins are a class of polypeptide antibiotics derived from *Paenibacillus polymyxa*, commonly used to treat infections caused by Gram-negative bacteria [7]. Similarly, daptomycin, a cyclic lipopeptide produced by *Streptomyces roseosporus*, is developed for the treatment of infections caused by susceptible and resistant Gram-positive bacteria [8]. Genomic technology and big data have provided powerful tools for utilizing genetic information to explore the genetic sequences and functions of soil bacteria. Bacterial genomes represent an essential resource for unlocking valuable information from the blueprint of living organisms [9]. Genomic data can reveal functional genes responsible for antimicrobial production, particularly through the identification of biosynthetic gene clusters (BGCs). These discoveries offer significant potential for applications in the biotechnology industry and pharmaceutical development [10].

This research focuses on exploring potential bacterial strains capable of producing AMP with the ability to inhibit specific pathogenic bacteria. This study included the purification and structural analysis of the AMP, along with proposing its mechanism of action against globally significant pathogens. Taxonomic identification and functional gene analysis will be performed using genomic data and bioinformatic tools, aiming to identify the BGCs responsible for AMP production. This work seeks to advance the understanding of novel AMPs and to establish a foundation for the development of new antimicrobial agents for future preclinical and clinical studies.

## 2. Results

### 2.1. Isolation and Screening of Soil-Derived Bacteria for Antibacterial Activity

**Figure 1:**
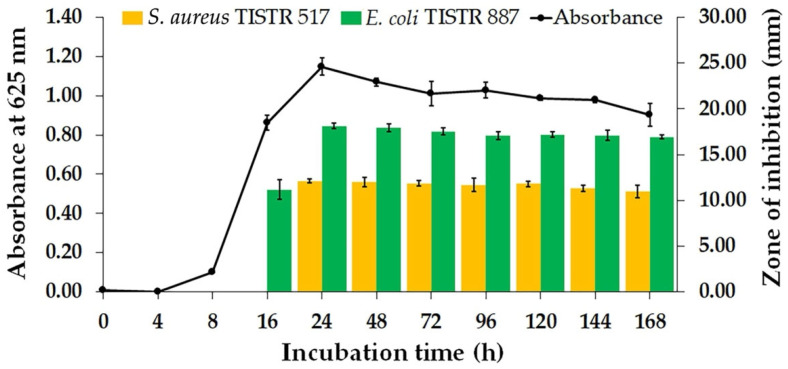
Bacterial growth curve and kinetics of antibacterial substance production by bacterial isolate Na14.

Bacterial strains were isolated from soil samples collected in the south of Thailand. A preliminary study on the cross-streak and agar well diffusion assay revealed that only one isolate, designated Na14, exhibited inhibitory activity against *E. coli* TISTR 887 and *S. aureus* TISTR 517 (Figures S1 and S2). The production of bioactive compounds by this bacterial isolate was evaluated by monitoring its growth curve and antibacterial activity. The result showed that the lag phase was observed at the first 4 h of incubation, and the cell-free supernatant (CFS) exhibited no detectable antibacterial activity (Figure 1). The exponential and the stationary phases occurred between 4–24 h and 24–168 h of the incubation period, respectively. Notably, the Na14 isolate produced a significantly higher level of bioactive compounds, with maximal antibacterial activity observed at 24 h. At this point, coinciding with the onset of the stationary phase, the inhibition zones of the CFS were 18.12 ± 0.32 mm for *E. coli* TISTR 887 and 12.11 ± 0.24 mm for *S. aureus* TISTR 517. The CFS retained its antibacterial activity without significant variation between 48 and 168 h. Moreover, it was demonstrated that the active substances had higher efficacy against the tested Gram-negative bacteria compared to Gram-positive bacteria.

The results of the production kinetics study revealed that the Na14 isolate produced active compounds with maximal efficacy at 24 h of incubation, indicating this as the optimal incubation period. Subsequently, the CFS obtained at 24 h of cultivation was prepared and evaluated for its antimicrobial activity against additional pathogenic bacteria (Table 1). The bioactive compounds from the Na14 strain exhibited significantly larger inhibition zones against Gram-negative bacteria, ranging from 18.19 ± 0.46 to 18.44 ± 0.26 mm, compared to Gram-positive bacteria, which ranged from 11.33 ± 0.41 to 12.45 ± 0.73 mm. These findings indicated that the Na14 strain predominantly produced compounds with higher efficacy against Gram-negative bacteria. Moreover, colistin and vancomycin were used as positive controls for Gram-negative and Gram-positive bacteria, respectively. The zones of inhibition ranged from 26.52 ± 0.43 to 29.41 ± 0.65 mm for colistin and 28.50 ± 0.22 to 28.96 ± 0.43 mm for vancomycin.

### 2.2. Purification of Bioactive Compounds of the Na14 Isolate

**Figure 2:**
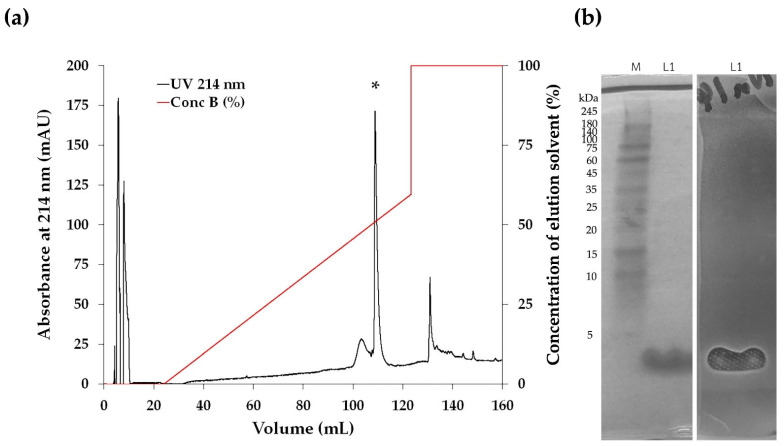
(**a**) Reversed-phase HPLC chromatogram of bioactive compounds obtained from the Na14 isolate. The purified fraction was obtained by RPC. Each peak fraction was evaluated for antimicrobial activity against *E. coli* TISTR 887 using the agar well diffusion method. An asterisk (*) indicates the peak fractions containing the active compounds. (**b**) SDS-PAGE and gel overlay inhibition assay of the purified Na14 peptide. (Left) Peptides obtained from RPC (L1) were analyzed using 15% SDS-PAGE and visualized by Coomassie blue staining. A protein marker (M) was included for molecular weight comparison. (Right) The SDS-PAGE gel was overlaid with soft agar containing *E. coli* TISTR 887 and incubated at 37 °C for 18 h. An inhibition zone corresponding to the Na14 peptide was observed.

The antimicrobial compounds in the CFS of the Na14 isolate were initially purified using ammonium sulfate precipitation. The fractions obtained at 50–75% saturation exhibited the greatest inhibition zone against *E. coli* TISTR 887. These active fractions were further purified using cation-exchange chromatography (CIEX), followed by reversed-phase chromatography (RPC). A single peak corresponding to the bioactive compounds was observed during RPC when the concentration of the elution solvent reached 51% (Figure 2a). A purification balance was compiled to quantitatively assess the efficiency at each stage of the purification process (Table 2). As the purification process progressed, the total amount of active peptides decreased, while their specific activity increased. After salt precipitation, the resulting fraction exhibited a specific activity of 60.31 AU/mg, representing a 3.23-fold increase in purity. The specific activity of the Na14 peptide increased from 100.63 to 835.99 AU/mg following sequential purification by CIEX and RPC. Additionally, the peptide purity increased by 5.38-fold and 44.73-fold after each respective purification step.

The purified peptide was subsequently analyzed using 15% SDS-PAGE to estimate its molecular weight. The results showed that fractions with purified active compounds obtained from RPC exhibited a peptide band with a molecular weight below 5 kDa. The corresponding gel was subsequently overlaid with soft agar containing *E. coli* TISTR 887. An inhibition zone was observed at the same position as the peptide band, indicating that the Na14 peptide exhibited antimicrobial activity (Figure 2b). These findings suggest that the purification process effectively separated the Na14 peptide while retaining its antimicrobial activity. Consequently, the purified peptide was subjected to further characterization.

### 2.3. Characterization of the Na14 Peptide

**Figure 3:**
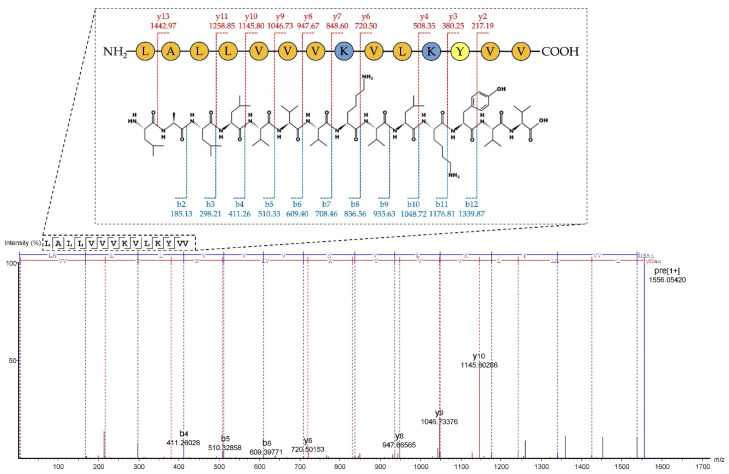
The *de novo* sequencing of the Na14 peptide by LC-MS/MS. The fragmentation patterns corresponding to b- and y-ion series were observed, and the resulting mass differences were analyzed to reconstruct the amino acid sequence.

The purified Na14 peptide obtained from RPC was analyzed using liquid chromatography–tandem mass spectrometry (LC-MS/MS). The peptides were ionized and subsequently fragmented into several product ions, especially b- and y-ions at peptide bonds. The amino acid sequence was determined using a *de novo* sequencing approach. The results showed that the Na14 peptide consisted of 14 amino acid residues with a molecular mass of 1555.05 Da. The peptide sequence was identified as LALLVVVKVLKYVV with an average local confidence (ALC) score of 78% (Figure 3). The physicochemical properties of the Na14 peptide were predicted using ProtParam (https://web.expasy.org/protparam; accessed on 26 June 2025) [11]. The predicted isoelectric point (pI) was 9.70. The presence of two lysine (K) residues contributed to a net positive charge of +2 at pH 7.4. The abundance of hydrophobic amino acids, such as valine (V), leucine (L), tyrosine (Y), and alanine (A), resulted in a high hydrophobicity index of 0.958 and a hydrophobic moment of 0.249. It indicated that the Na14 peptide possessed amphipathic characteristics. Moreover, this peptide had the predicted instability index of –4.90, suggesting that it is likely to be stable under *in vivo* conditions [12].

**Figure 4:**
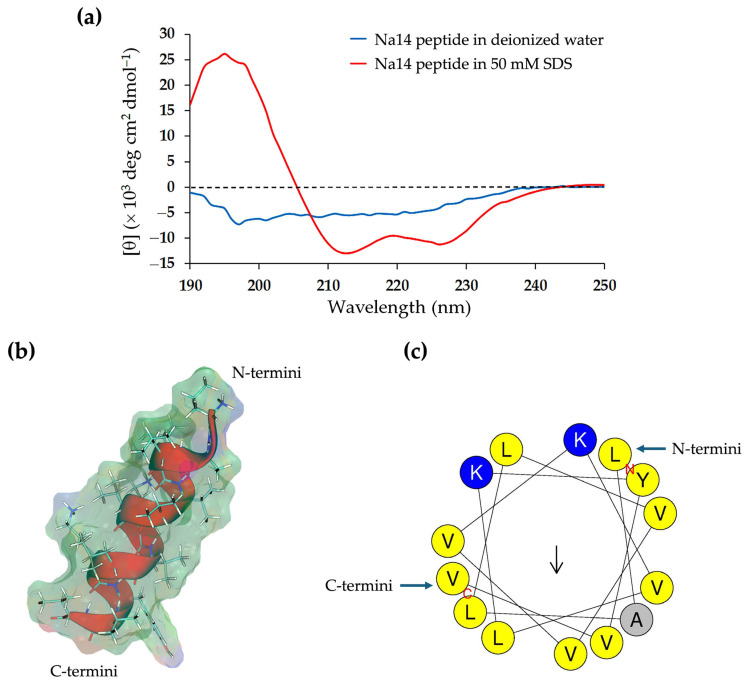
(**a**) Circular dichroism spectra of the Na14 peptide. The peptide was dissolved in either deionized water (blue) or a 50 mM SDS solution (red). (**b**) Structural modeling of the Na14 peptide using AlphaFold. The peptide structure is shown in cartoon style with side chains displayed as stick-and-ball models and overlaid with the molecular surface. The surface is color-coded to indicate polarity: blue for positively charged regions, red for negatively charged regions, and green for non-polar areas. (**c**) Predicted α-helical conformation of the Na14 peptide, generated by HeliQuest. The helical wheel projection illustrates the amphipathic nature of the peptide using single-letter amino acid codes and color shading: blue for polar residues, yellow for non-polar residues, and grey for small residues.

The content of the secondary structure of the Na14 peptide was analyzed based on its circular dichroism (CD) spectrum (Figure 4a). When dissolved in deionized water, the peptide exhibited a negative CD band near 200 nm and low ellipticity above 235 nm, indicating a predominance of random coil (56.8%) and turn (23.7%) conformations (Table 3). In contrast, in the presence of SDS micelles served as a membrane-mimicking agent, the CD spectrum displayed a positive band at 195 nm and two negative bands around 210 nm and 222 nm, suggesting an increase in α-helical content (24.4%) and a corresponding decrease in the proportion of random coil structure (43.9%). The structural model of the Na14 peptide was generated using AlphaFold, revealing that the peptide adopted the α-helical conformation (Figure 4b). The helical wheel projection produced using HeliQuest (https://heliquest.ipmc.cnrs.fr/index.html; accessed on 26 June 2025) indicated that the polar amino acids were positioned on one side of the helix, while the non-polar residues were located on the opposite side (Figure 4c).

### 2.4. Antimicrobial Activity of the Na14 Peptide

The antimicrobial activity of the purified Na14 peptide was evaluated using the broth microdilution assay. The results demonstrated that the Na14 peptide exhibited strong activity against Gram-negative bacteria, including *E. coli* TISTR 887, *P. aeruginosa* TISTR 357, and *K. pneumoniae* TISTR 1383, with minimum inhibitory concentration (MIC) and minimum bactericidal concentration (MBC) values ranging from 2 to 4 µg/mL (Table 4). In contrast, the peptide was ineffective against Gram-positive bacteria such as *S. aureus* TISTR 517 and MRSA strain 2468, with MIC and MBC values exceeding 128 µg/mL. Furthermore, the antimicrobial potency of the Na14 peptide against Gram-negative bacteria was comparable to that of colistin, which exhibited MIC and MBC values between 1 and 2 µg/mL. In comparison, vancomycin showed superior efficacy against Gram-positive bacteria, with MIC and MBC values of 2 and 4 µg/mL, respectively.

### 2.5. Time–Kill Kinetics of the Na14 Peptide

**Figure 5:**
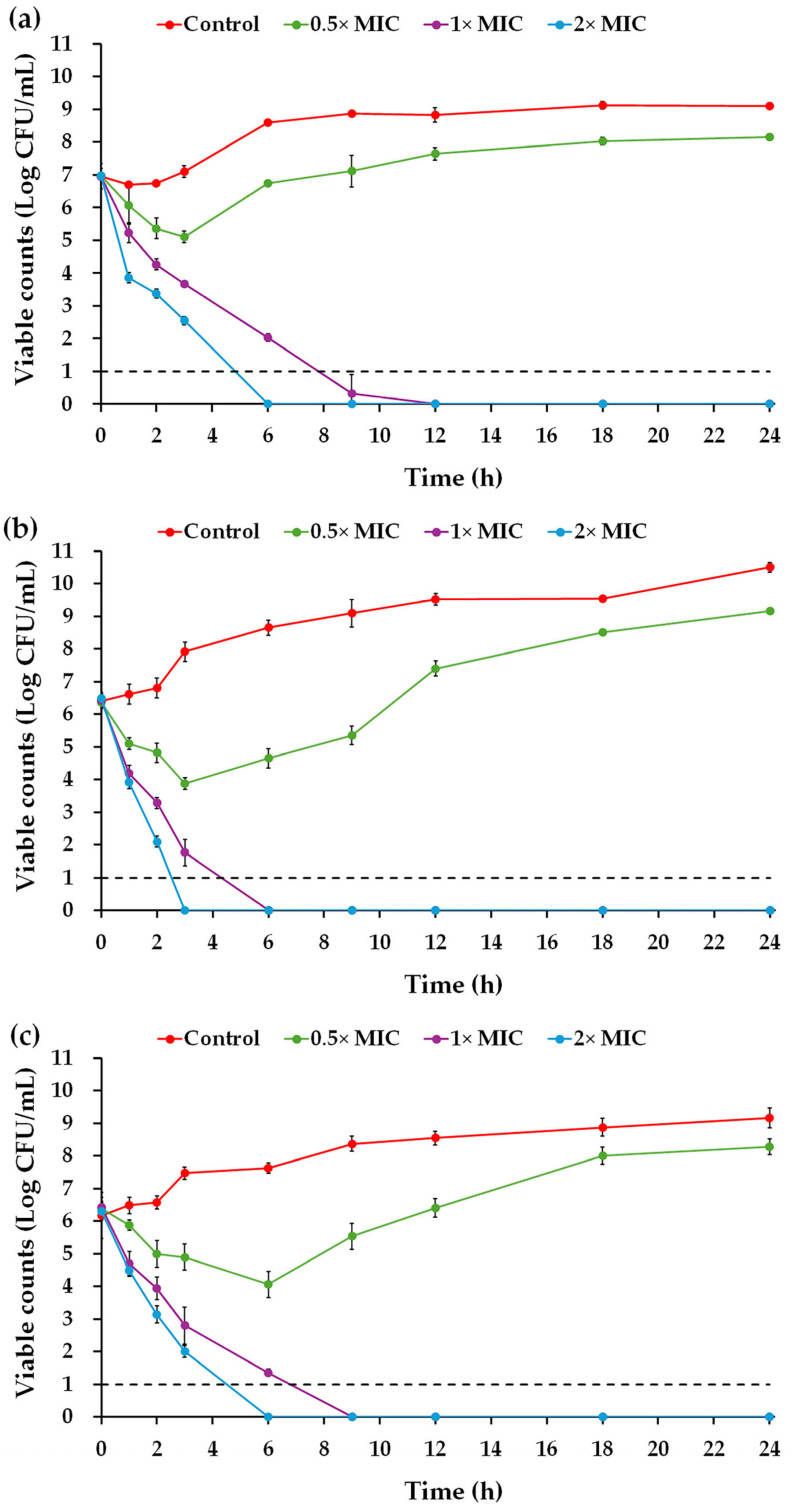
Time–kill kinetics of the Na14 peptide. The Na14 peptides at different concentrations (0.5×, 1×, and 2× MIC) were tested against (**a**) *E. coli* TISTR 887, (**b**) *P. aeruginosa* TISTR 357, and (**c**) *K. pneumoniae* TISTR 1383. Untreated cells served as controls. At specified time intervals, either undiluted or diluted bacterial suspensions were plated on MH agar. Viable cell counts were determined and expressed on a logarithmic scale. The limit of detection (LOD) of the assay was 10 CFU/mL, indicated by a dashed line.

The time–kill curve demonstrated that the Na14 peptide at 0.5× MIC temporarily suppressed the growth of *E. coli* TISTR 887 and *P. aeruginosa* TISTR 357 during the first 3 h of incubation (Figure 5a,b), while the growth of *K. pneumoniae* TISTR 1383 was inhibited for up to 6 h (Figure 5c). Thereafter, all bacterial strains resumed growth, which continued to increase until the end of the 24 h observation period. In contrast, treatment with higher peptide concentrations effectively killed all tested bacteria. Specifically, the peptide at 1× MIC eliminated *E. coli* TISTR 887, *P. aeruginosa* TISTR 357, and *K. pneumoniae* TISTR 1383 at 12, 6, and 9 h, respectively. Furthermore, the peptide at 2× MIC completely eradicated *E. coli* TISTR 887 and *K. pneumoniae* TISTR 1383 at 6 h and *P. aeruginosa* TISTR 357 at 3 h. The killing rate of the Na14 peptide was calculated to evaluate its effect on cell reduction. The killing rates of the peptide at 1× MIC were 1.33 ± 0.07, 2.47 ± 0.09, and 1.64 ± 0.05 h^−1^ against *E. coli* TISTR 887, *P. aeruginosa* TISTR 357, and *K. pneumoniae* TISTR 1383, respectively. When treated with 2× MIC of Na14 peptide, the corresponding killing rates increased to 2.68 ± 0.09, 4.99 ± 0.12, and 2.42 ± 0.10 h^−1^, respectively. These results indicate that higher concentrations of the peptide enhance the bacterial killing rate.

### 2.6. Morphological Changes of Bacterial Cells Induced by the Na14 Peptide

**Figure 6:**
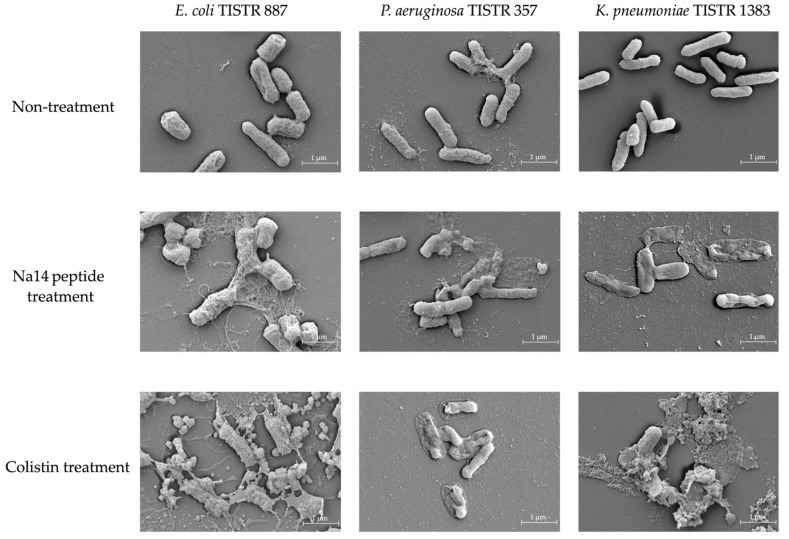
SEM images showing morphological changes in bacterial cells following treatment with the Na14 peptide and colistin at 1× MIC level, compared to the untreated control. Images were captured at 20,000× magnification.

The morphologies of *E. coli* TISTR 887, *P. aeruginosa* TISTR 357, and *K. pneumoniae* TISTR 1383 following treatment with the Na14 peptide were examined and compared to those treated with colistin at the same concentration (1× MIC) using a scanning electron microscopy (SEM). In the untreated groups, all bacterial strains retained their rod-shaped morphology, indicating intact cell membranes (Figure 6). After 16 h of exposure to the Na14 peptide, cytoplasmic extrusion was observed, suggesting membrane disruption and signs of cell lysis. Similarly, cells treated with colistin also exhibited apparent evidence of lysis. However, the extent of cell lysis appeared to be more evident in the colistin-treated group compared to those treated with the Na14 peptide.

### 2.7. Effect of Temperature, pH, and Enzyme on Peptide Stability

The stability of the purified Na14 peptide was evaluated under various conditions, including heat exposure, enzymatic digestion, and pH variation. A concentration of 10× MIC of Na14 peptide was used to assess its antimicrobial activity against the tested pathogens. The remaining activity of the treated samples was compared to that of the untreated control (Table 5). Thermal stability analysis revealed that the peptide retained its antimicrobial activity against all pathogens across a temperature range of 30 °C to 90 °C, with the residual activity ranging from 97.22 ± 1.62% to 100.89 ± 0.39%. The peptide also demonstrated tolerance to a broad pH range between pH 3 and 11, maintaining activity between 97.62 ± 2.08% and 100.85 ± 1.95% throughout the study. Enzymatic stability was evaluated using various proteases. Among these, trypsin treatment exhibited the most significant impact, markedly reducing the antimicrobial activity of the Na14 peptide across all tested bacterial strains, with reductions ranging from 88.72 ± 0.75% to 89.53 ± 1.30%. Treatments with α-chymotrypsin or proteinase K significantly caused moderate reductions in the Na14 peptide activity between 93.23 ± 2.60% and 96.50 ± 1.56%. Furthermore, no significant differences in residual activity were observed across the tested pathogens under any condition, indicating that the Na14 peptide retained consistent antimicrobial activity regardless of the bacterial species.

### 2.8. Genome Analysis and Bacterial Identification

**Figure 7:**
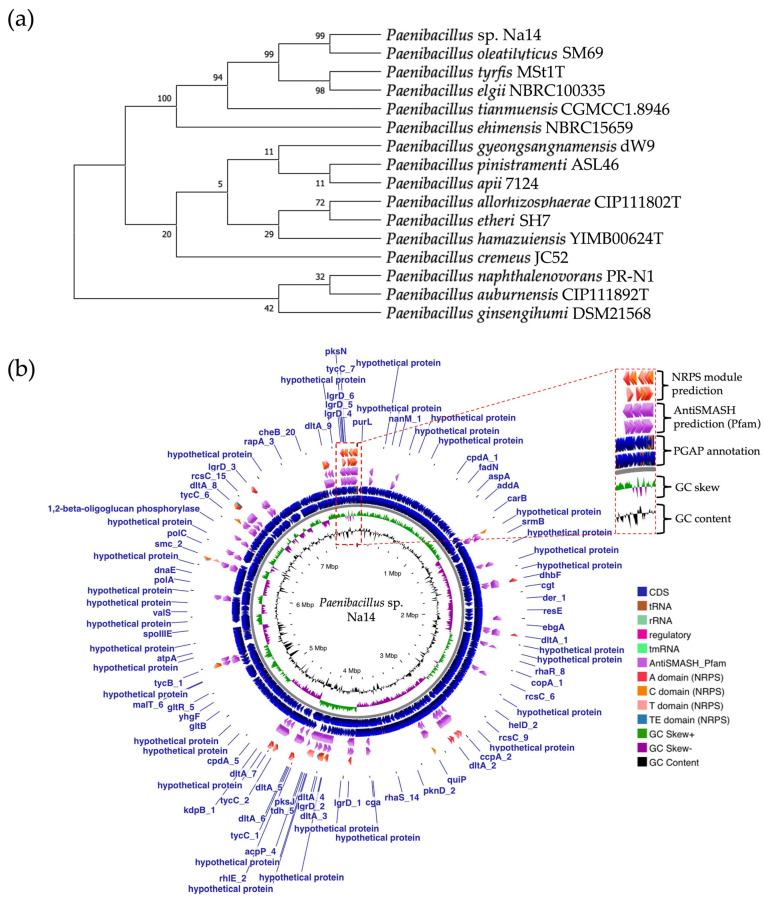
Genomic identification and taxonomic classification of *Paenibacillus* sp. Na14. (**a**) The phylogenetic tree illustrates the relationship of the Na14 strain to other members of the *Paenibacillus* genus, showing the closest taxonomic affiliation to *Paenibacillus oleatilyticus* SM69. (**b**) The circular genome map displays annotated genes along with predicted biosynthetic gene clusters associated with secondary metabolite production. NRPS-encoding regions and their domains are visualized in detail.

A single colony of the Na14 strain was observed under a light microscope at 1000× magnification, revealing Gram-positive, rod-shaped cells with endospore-forming capability. The genomic sequence of the Na14 strain was obtained using the Illumina HiSeq platform. Paired-end raw reads were quality-checked using FastQC, followed by *de novo* assembly using SPAdes (version 3.14.1). The assembled genome demonstrated a completeness of 99.14% with a sequencing coverage of 236×. The result revealed a circular genome comprising 7,789,831 base pairs, deposited under the accession number CP186533 at the National Center for Biotechnology Information (NCBI). Species identification was performed using the Type (Strain) Genome Server (TYGS) and constructed phylogenetic tree with MEGA X software (version 10.1.8). The Na14 strain showed the highest similarity to *Paenibacillus oleatilyticus* SM69, with digital DNA–DNA hybridization (dDDH) values of 79.1% (95% CI: 75.1–82.5) for formula d_0_, 61.0% (95% CI: 58.1–63.8) for formula d_4_, and 78.1% (95% CI: 74.7–81.2) for formula d_6_ (Figure 7a). Taxonomic classification placed the Na14 strain within the genus *Paenibacillus*, but the dDDH value for formula d_4_ remained below the 70% threshold, suggesting that Na14 may represent a novel species. The circular genome map of the Na14 strain was visualized using the Proksee platform (CGViewBuilder version 2.0.5, https://proksee.ca, accessed on 10 March 2025), based on gene annotations provided by the NCBI Prokaryotic Genome Annotation Pipeline (PGAP). The annotated genome comprised a total of 7130 genes, including 7054 coding DNA sequences (CDSs) and 76 RNA genes. The RNA genes consisted of 3 rRNAs, 69 tRNAs, and 4 non-coding RNAs (ncRNAs). The potential for secondary metabolite production was assessed using the Antibiotics and Secondary Metabolite Analysis Shell (antiSMASH; version 7.0). A total of 27 biosynthetic gene clusters (BGCs) were identified, including 4 terpene clusters, 1 opine-like metallophore cluster, 11 non-ribosomal peptide synthetase (NRPS) clusters, 7 polyketide synthase (PKS) clusters, 2 ribosomally synthesized and post-translationally modified peptide (RiPP) clusters, and 2 cyclic lactone auto-inducer clusters. The non-ribosomal peptide synthetase (NRPS) modules identified in the genome of the Na14 strain were composed of adenylation (A), condensation (C), thiolation (T), and thioesterase (TE) domains. These domains were visualized on the circular genome map to illustrate the distribution of NRPS-related genes (Figure 7b). In addition, antibiotic resistance genes (ARGs) were identified by comparing the annotated genome against reference sequences from the Comprehensive Antibiotic Resistance Database (CARD) (https://card.mcmaster.ca, accessed on 10 March 2025). A total of 11 antimicrobial resistance genes were detected, including 8 genes associated with glycopeptide resistance, exhibiting 33.75–36.90% identity and 55.48–133.51% coverage; 1 gene related to phenicol resistance, showing 68.06% identity and 99.09% coverage; 1 gene conferring cephalosporin resistance, with 67.45% identity and 100.00% coverage; and 1 gene involved in antibiotic efflux, displaying 90.67% identity and 100.00% coverage. The prediction of antimicrobial production-related genes and antimicrobial resistance genes supports the hypothesis that the Na14 strain possesses both antimicrobial biosynthesis capabilities and self-defense mechanisms.

### 2.9. Comparative Analysis of NRPS-Encoding Biosynthetic Gene Clusters Involved in AMP Production

Biosynthetic gene clusters (BGCs) encoding non-ribosomal peptide synthetases (NRPS) play a crucial role in determining the AMP production. The Na14 peptide showed a similarity of amino acid sequence to brevibacillin 2V, produced by the NRPS BGCs of *Brevibacillus laterosporus* DSM 25 [13]. These findings support further in-depth investigation of the NRPS modules in the Na14 strain to characterize the BGCs involved in AMP biosynthesis and to propose their functional roles in AMP production by *Paenibacillus* sp. Na14. The amino acid sequences obtained from genomic analysis of NRPS modules by antiSMASH were modeled using the AlphaFold Server (https://alphafoldserver.com, accessed on 20 March 2025). The predicted protein structures were compared to known reference structures using FoldSeek (https://search.foldseek.com, accessed on 20 March 2025), and the best-matching structural homologs were identified based on sequence identity and template modeling (TM) scores. The NRPS architecture was proposed to consist of 1 initial module containing A and T domains, followed by 12 elongation modules composed of C, A, and T domains, and 1 termination module comprising C, A, T, and TE domains. The initial NRPS module in *Paenibacillus* sp. Na14 exhibited structural similarity to the thiolation-state module of linear gramicidin synthetase subunit A from *Brevibacillus parabrevis* (PDB ID: 5ES8), with a sequence (seq.) identity of 39.5% and a TM score of 0.83386. The elongation process of the peptide synthesis was predicted to involve 12 modules. It shared structural similarity with the condensation-state module of the same enzyme from *Brevibacillus parabrevis* (PDB ID: 6MFZ), with the seq. identities ranging from 31.9% to 40.2% and TM-scores between 0.74125 and 0.88822. It was also similar to the termination module of surfactin A synthetase C from *Bacillus subtilis* (PDB ID: 2VSQ), exhibiting seq. identities ranging from 34.0% to 41.7% and TM-scores between 0.46144 and 0.77661. The termination module of Na14-synthesizing enzyme showed structural similarity to the termination module of surfactin A synthetase C (PDB ID: 2VSQ), with a seq. identity of 33.7% and a TM-score of 0.63631. These structural similarities are supported by the taxonomic classification of *Paenibacillus* sp. Na14 and *Brevibacillus parabrevis*, which both belong to the family Paenibacillaceae. Moreover, *Paenibacillus* sp. Na14 and *Bacillus subtilis* are members of the order Bacillales. Notably, one of the elongation modules also showed 27.0% seq. identity and a TM-score of 0.77945 to the serine module of the teixobactin-producing NRPS Txo2 from *Eleftheria terrae* (PDB ID: 6P1J), a bacterium from a distinct phylum (Pseudomonadota). The observed structural overlap between the predicted NRPS modules of the Na14 peptide and known NRPS structures in the Protein Data Bank (PDB) supports a conserved topological organization, suggesting their involvement in antimicrobial peptide biosynthesis within the genome of *Paenibacillus* sp. Na14 (Table 6).

## 3. Discussion

Bacterial infections continue to pose a significant global health threat, particularly those caused by antibiotic-resistant strains [14]. Recently, the WHO has revised the list of bacterial priority pathogens in 2024 to guide investments in the development of new and effective antibiotics and to support public health strategies for the surveillance and control of antimicrobial resistance [15]. Several Gram-negative pathogens exhibiting intrinsic or acquired antibiotic resistance are classified among the highest priority levels. For example, *E. coli* and *K. pneumoniae* are categorized as critical priority pathogens, while *P. aeruginosa* is listed as a high-priority group. Numerous research sectors are actively seeking novel antimicrobial agents, with particular emphasis on those derived from soil bacteria.

In this study, a soil bacterium designated as Na14 was isolated and found to exhibit antimicrobial activity against *E. coli* TISTR 887 and *S. aureus* TISTR 517. The Na14 isolate produced bioactive compounds exhibiting maximal antimicrobial activity at 24 h of incubation. This time point corresponded to the early stationary phase, a stage in which bacteria commonly produce secondary metabolites, such as antibiotics or antimicrobial peptides, due to various physiological and ecological factors [16]. For example, bacitracin produced by *Bacillus paralicheniformis* began the synthesis after 5 h of incubation, and its production level was high after 8–10 h of growth, corresponding to the onset of the stationary phase [17]. In addition, the production of surfactin lipopeptide by *Bacillus velezensis* SK was first detected at the mid-exponential phase at 16 h. Surfactin production reached its maximum level by the end of the stationary phase at 36 h, likely due to nutrient-depleted conditions [18].

The bioactive compound from the Na14 strain was identified as an AMP with the amino acid sequence of LALLVVVKVLKYVV. This peptide exhibited amphipathic characteristics, comprising a cationic region and a hydrophobic domain. Additionally, the Na14 peptide was classified as a valine-rich (42.9%) and leucine-rich (28.6%) AMP, as the content of each amino acid exceeded 25% [19]. The high abundance of these hydrophobic amino acids might exhibit antimicrobial properties through their hydrophobic nature and membrane-disruptive capabilities. A previous study analyzing AMPs in the Antimicrobial Peptide Database (APD) reported that 21% of AMPs met this criterion of amino acid richness. Notably, leucine- and valine-rich AMPs, such as gramicidins B and C, were common among effective AMPs from bacteria [19]. The Na14 peptide was a novel AMP because its sequence was not included in the Database of Antimicrobial Activity and Structure of Peptides (DBAASP) (https://dbaasp.org/search; 23,036 peptides; accessed on 23 June 2025) and the Antimicrobial Peptide Database (APD) (https://aps.unmc.edu/database; 5513 peptides; accessed on 23 June 2025). The Na14 peptide sequence was aligned with other AMPs in the APD database. The highest sequence similarity (64.29%) was observed with brevibacillin 2V from *Brevibacillus laterosporus* DSM 25 (APD ID: AP03330) [13].

The CD spectrum indicated that the Na14 peptide in water predominantly adopted random coil and turn structures. SDS micelles, commonly used as membrane-mimicking agents, provide a hydrophobic environment that simulates the lipid bilayer of cell membranes. These micelles are often employed to assess changes in peptide conformation and interactions, thereby offering insights into peptide-membrane interactions [20]. In the presence of SDS micelle, the Na14 peptide underwent a conformational transition characterized by an increase in α-helical content, as reflected in its distinct CD spectrum. It is presumed that the Na14 peptide adsorbs onto the micellar surface and undergoes a conformational transition from a random coil to an α-helix [21]. Similar conformational changes in certain AMPs have been reported in previous studies. For example, brevibacillin initially exhibited a random coil structure but underwent a transition to an α-helical structure (43.14%) upon the addition of SDS [21]. This suggested that brevibacillin disrupted the cytoplasmic membrane of bacterial cells, ultimately leading to cell death. In addition, brevibacillin 2V was found to bind to the cell wall synthesis precursor Lipid II, inducing a significant conformational change characterized by an increased α-helical content [13]. Brevibacillin 2V also caused bacterial membrane permeability, supporting its bactericidal mechanism of action. Therefore, the structural transition of the Na14 peptide to an α-helical conformation may represent a mechanism underlying its activity, which requires further characterization.

The Na14 peptide demonstrated potent antimicrobial activity against Gram-negative bacteria, including *E. coli* TISTR 887, *P. aeruginosa* TISTR 357, and *K. pneumoniae* TISTR 1383. Its activity was comparable to that of colistin, exhibiting a bactericidal effect at its 1× MIC level. Brevibacillin 2V, a structurally similar peptide derived from *Brevibacillus laterosporus* DSM 25, has been reported to display broad-spectrum antimicrobial activity against both Gram-negative and Gram-positive bacteria. Notably, brevibacillin 2V showed MIC values of 16 µg/mL against *E. coli* ATCC 25922, 64 µg/mL against *P. aeruginosa* LMG6395, 32 µg/mL against *K. pneumoniae* LMG20218, and 2 µg/mL against MRSA ATCC 15975 [13]. The time–kill kinetic assay indicated that the Na14 peptide at 1× MIC exerted bactericidal activity, reducing bacterial counts by more than 3 log units. Its efficacy was concentration-dependent, with higher peptide concentrations resulting in an increased bacterial killing rate. The Na14 peptide at 2× MIC completely killed *E. coli* TISTR 887 and *K. pneumoniae* TISTR 1383 within 6 h, and *P. aeruginosa* TISTR 357 within 3 h. In comparison, previous studies have shown that brevibacillin 2V at 10× MIC achieved complete killing of MRSA within 6 h, and a similar killing kinetics was observed for nisin [22]. These findings suggested that certain brevibacillins acted as fast-acting bactericidal peptides through disruption of the bacterial membranes. Additionally, brevicidine derived from *Brevibacillus laterosporus* DSM25 rapidly eradicated *E. coli* in 1 h when treated with the peptide at 4× MIC. In contrast, the pathogen remained viable at lower concentrations of brevicidine, ranging from 0.5× to 2× MIC. Brevicidine at higher concentrations exhibited its bactericidal activity by disrupting the outer membrane of Gram-negative bacteria, thereby facilitating its access to and subsequent interaction with inner membrane components [23]. SEM analysis revealed membrane damage in bacterial cells treated with either Na14 peptide or colistin at 1× MIC, showing clear morphological changes such as cell rupture and lysis. Previous SEM studies demonstrated that several AMPs targeted the bacterial membrane by forming pores and causing the extrusion of cellular contents, indicating membrane damage [24,25,26]. The Na14 peptide exhibited a MIC equal to its MBC, indicating bactericidal activity at 1× MIC. Its proposed mechanism involves the positively charged lysine residues facilitating electrostatic binding to the negatively charged bacterial cell wall and membrane. The hydrophobic regions then enable insertion into the lipid bilayer, leading to pore formation and subsequent cell lysis. However, the specific molecular interactions with membrane-integrated components remain to be elucidated and will be the focus of future studies.

Peptide stability is a critical factor influencing the effectiveness of AMP. The Na14 peptide demonstrated strong thermal tolerance across a wide temperature range (30–90 °C). The Na14 peptide is a linear AMP with an amino acid sequence similar to members of the brevibacillin and brevilaterin families, which have been previously isolated from *Brevibacillus laterosporus* [27]. Previous studies have reported that brevibacillins and brevilaterins exhibit excellent thermal stability, maintaining their antimicrobial activity across a wide temperature range from 40 °C to 100 °C [28,29]. The acid–base stability of Na14 peptide showed broad pH tolerance ranging from pH 3 to 11. In comparison, the structurally related peptides, such as brevibacillin, have been reported to remain stable between pH 5 and 7, while brevibacillin V demonstrated stability over a pH range of 5 to 9 [28]. Proteolytic enzymes pose a significant challenge to the efficacy of AMPs by cleaving peptide bonds. These peptide bonds form the structural backbone of AMPs and are essential for maintaining their stability and function. The Na14 peptide exhibited partial degradation by trypsin, α-chymotrypsin, and proteinase K, while retaining over 88% of its antimicrobial activity. Similarly, partial reductions in activity due to protease susceptibility have been reported for brevilaterins derived from *Brevibacillus laterosporus* S62-9 and certain AMPs produced by *Brevibacillus laterosporus* strains BGSP7, BGSP9, and BGSP11, indicating comparable enzymatic sensitivity to that observed in the Na14 peptide [30,31]. The overall activity of the Na14 peptide under various conditions demonstrated that it is a stable AMP, providing strong evidence for its potential in further pharmacokinetic studies and formulation development.

The Na14 strain was identified as *Paenibacillus* sp. Na14. *Paenibacillus* spp. are Gram-positive, endospore-forming bacteria capable of surviving in harsh environmental conditions. Genomic analyses have identified several BGCs that are crucial for the production of diverse antimicrobial compounds, many of which show potential for clinical application in the treatment of antimicrobial-resistant infections. Among these, bacteriocins, a class of AMPs produced through ribosomal synthesis, represent an important group of bioactive compounds [32]. Several studies have reported potent antimicrobial agents from *Paenibacillus* spp., such as *Paenibacillus polymyxa* OSY-DF, *Paenibacillus polymyxa* NRRL B-30509, and *Paenibacillus* sp. strain A3, producing paenibacillin, paenicidin A, and penisin, respectively [33,34,35]. *Paenibacillus* spp. also produced various AMPs through NRPS system, including members of the polymyxin family such as colistin. These compounds are well-known for their clinical use in treating antibiotic-resistant Gram-negative bacterial infections. Notably, polymyxins have been identified in *Paenibacillus polymyxa*, *Paenibacillus alvei*, and *Paenibacillus kobensis* [36,37]. A truncated structural variant of polymyxins, known as octapeptins, has been reported to exhibit antimicrobial activity against both Gram-positive and Gram-negative bacteria, while demonstrating low toxicity to mammalian cells [38]. In addition to the various BGCs of antimicrobial compounds found in *Paenibacillus* spp., natural resistance to self-produced antimicrobials plays a vital role in enabling the bacteria to tolerate their antimicrobial compounds. Numerous genes related to antimicrobial resistance have been reported in their genomic data, including ABC transporters and genes conferring resistance to both broad and specific classes of antibiotics. Resistance genes, identified in some *Paenibacillus* spp. including *Paenibacillus* sp. Na14, targeted lipopeptide antibiotics, penicillins, and cephalosporins, supporting the idea that these resistance mechanisms serve as self-defense systems against their antimicrobial products [39]. The genomic analysis of *Paenibacillus* sp. Na14 revealed the presence of 27 BGCs associated with secondary metabolism, including those responsible for the production of bacteriocins and NRPS-synthesizing AMPs. Numerous studies found genes related to secondary metabolite biosynthesis in various *Paenibacillus* species, supporting the functional potential observed in the Na14 strain. Interestingly, the amino acid sequence of the purified AMP isolated from the Na14 culture showed high similarity to that of brevibacillin 2V, a lipopeptide produced by *Brevibacillus laterosporus* DSM 25 [13]. *Brevibacillus* spp. are Gram-positive, endospore-forming bacteria that belong to the same taxonomic family of Paenibacillaceae, including *Paenibacillus* spp. AMP production in *Brevibacillus* spp. is predominantly mediated by NRPS systems. Several AMPs have been reported from *Brevibacillus* spp., including linear gramicidin A, B, and C, which are composed of 15 amino acid residues and are synthesized by 15 NRPS modules, each containing an A domain. The A domain is responsible for the selection and incorporation of specific amino acids during peptide chain synthesis. Linear gramicidin, originally isolated from *Brevibacillus brevis*, was one of the first AMPs successfully applied in a clinical setting [40,41]. Another group of AMPs produced by *Brevibacillus* spp. consists of NRPS-synthesized linear lipopeptides. These have been isolated from various species, including bogorols from *Brevibacillus laterosporus*, BT peptides from *Brevibacillus texasporus*, and brevibacillin 2V from *Brevibacillus laterosporus* DSM 25 [42,43,44]. The NRPS module responsible for AMP production is mostly found in *Paenibacillus* spp. and *Brevibacillus* spp. [39]. The structural similarity observed between the NRPS modules and AMP products of *Paenibacillus* sp. Na14 and *Brevibacillus* spp. supports the presence of conserved homologous features. This similarity is further reinforced by the genotypic and taxonomic relationships shared between the two genera. The evolutionary connection at the family level (Paenibacillaceae) suggests a common ancestral origin, supporting the proposed homology in genetic sequences, encoded proteins, and secondary metabolite biosynthetic pathways [45]. The similarity in protein structure homology between the two genera may originate from horizontal gene transfer, as previously reported in other studies investigating shared antibiotic resistomes between *Paenibacillus* spp. and *Brevibacillus* spp. [39].

## 4. Materials and Methods

### 4.1. Materials

Ammonium sulfate, sodium acetate, sodium chloride (NaCl), sodium dodecyl sulfate (SDS), sodium hydroxide (NaOH), hydrochloric acid (HCl), methanol, ethanol, and acetonitrile (ACN) were supplied by RCI Labscan Ltd., Bangkok, Thailand. Mueller–Hinton (MH) agar and Luria Bertani (LB) broth were obtained from Titan Biotech Ltd., Rajasthan, India. Colistin, vancomycin, and Coomassie brilliant blue G-250 were procured from Sigma Aldrich Inc., St. Louis, Missouri, USA. Glacial acetic acid and trifluoroacetic acid (TFA) were obtained from Merck KGaA, Darmstadt, Germany. *Staphylococcus aureus* TISTR 517, *Escherichia coli* TISTR 887, *Pseudomonas aeruginosa* TISTR 357, and *Klebsiella pneumoniae* TISTR 1383 were obtained from the Thailand Institute of Scientific and Technological Research (TISTR), Thailand. MRSA strain 142, 1096, and 2468 were provided by the Medical Technology Laboratory, School of Allied Health Sciences, Walailak University, Thailand.

### 4.2. Sample Collection and Bacterial Isolation

The samples were obtained from soils at a depth of 10–15 cm in the south of Thailand. They were placed in polyethylene bags and kept in an ice-cooled container. Subsequently, the samples were dried at 50 °C in a hot air oven for two days. The dried samples (10 g) were resuspended in 90 mL of sterile 0.9% NaCl solution. The soil suspension was shaken in an incubator at 150 rpm for 30 min under ambient conditions, followed by incubation at 60 °C for 30 min. Subsequently, the suspension was subjected to 10-fold serial dilutions up to 10^−6^. The diluted samples (100 µL) from each dilution were spread onto MH agar plates. The plates were incubated at 37 °C for one week, after which individual colonies were restreaked to obtain pure isolates [46].

### 4.3. Screening of Antibacterial Activity of Soil Bacteria

To identify potential active bacterial isolates, the cross-streak and agar well diffusion method was employed against *S. aureus* TISTR 517 and *E. coli* TISTR 887, following the protocol described in a previous study [47]. The production kinetics of antibacterial substances by the active bacterial isolates were investigated. Single colonies were suspended in 0.9% NaCl solution, and the turbidity was adjusted to an optical density (OD) of 0.35 at 625 nm. Two milliliters of the cell suspension were inoculated into 98 mL of LB broth and incubated at 37 °C with shaking at 150 rpm for seven days. At each time interval, bacterial growth was evaluated by measuring the OD at 625 nm using a UV-Vis spectrophotometer (JASCO Corporation, Tokyo, Japan). The cell-free supernatant (CFS) was obtained by centrifuging the culture at 10,000× *g* at 4 °C for 15 min. Subsequently, 100 µL of the supernatant was loaded into wells on MH agar plates precultured with tested pathogens [46]. Colistin and vancomycin served as positive controls in agar well diffusion assay.

### 4.4. Purification of the Bioactive Compound

Single colonies of the Na14 isolate were resuspended in 0.9% NaCl solution, and the cell suspension was adjusted to an OD of 0.35 at 625 nm. A 2% aliquot of this suspension was inoculated into 196 mL of LB broth and incubated at 37 °C for 24 h. The CFS was collected by centrifugation at 10,000× *g* for 15 min at 4 °C. Bioactive compounds were then precipitated using stepwise ammonium sulfate precipitation at 25%, 50%, and 75% saturation levels. The active compounds were purified using cation exchange chromatography (CIEX) followed by reversed-phase chromatography (RPC), as described in our previous study [48]. The purified compounds were concentrated using a refrigerated speed vacuum concentrator. The dried samples were reconstituted in deionized water and subjected to two-fold serial dilutions. An aliquot (100 µL) of each dilution was used to evaluate antimicrobial activity using the agar well diffusion method, employing *E. coli* TISTR 887 as the tested bacteria. The purification efficiency was assessed by calculating antimicrobial activity at each purification stage using the following equation [49].

$$\mathrm{Antimicrobial}\;\mathrm{activity}\;(\mathrm{arbitrary}\;\mathrm{unit}/\mathrm{mL};\;\mathrm{AU}/\mathrm{mL})=\;\frac{2^{n}\;\times \;1000}{V}$$

where n represents the reciprocal of the highest two-fold dilution that produces a visible inhibition zone, and V denotes the volume of the sample in µL.

### 4.5. Identification and Analysis of the Na14 Peptide

The purified Na14 peptide was reconstituted in a solvent containing 0.1% formic acid and 1% acetonitrile (ACN) and then analyzed using high-resolution liquid chromatography coupled with tandem mass spectrometry (LC-MS/MS) (Thermo Fisher Scientific Inc., Waltham, MA, USA). Peptide separation was carried out on a C18 reversed-phase column. The elution conditions and MS/MS fragmentation parameters followed the protocol described in our previous study [50]. Peptide identification was performed using Peak Studio X. Physicochemical properties, including molecular mass, isoelectric point (pI), net charge, and hydrophobicity, were predicted using the ProtParam and HeliQuest tools [11,51].

The peptides (5 mg) were subjected to analysis using a 15% sodium dodecyl sulfate-polyacrylamide gel electrophoresis (SDS-PAGE). The gel was electrophoresed at a constant voltage of 100 V. One half of the gel was stained using Coomassie brilliant blue G-250 to visualize protein bands, with a molecular weight marker included to facilitate band position comparison. The remaining half was fixed in a solution containing 5% acetic acid and 25% methanol for 30 min, followed by a 3 h wash with distilled water. Subsequently, 18 mL of soft MH agar, including *E. coli* TISTR 887, was overlaid onto the gel to assess antimicrobial activity by observing inhibition zones after incubation at 37 °C for 24 h [52].

To determine the secondary structure composition of the Na14 peptide, peptide samples (0.50 mg/mL) were prepared in either deionized water or 50 mM SDS solution [53]. Circular dichroism (CD) spectra were acquired using a J-815 spectropolarimeter (JASCO Corporation, Tokyo, Japan) at 25 °C over a wavelength range of 190–250 nm. Measurements were conducted in a 1 mm pathlength quartz cuvette. The instrument settings were as follows: a scanning speed of 100 nm/min, a data interval of 0.1 nm, and a bandwidth of 1.0 nm. Spectra were baseline-corrected by subtracting the signal of the corresponding diluent. Secondary structure estimation was performed using the CONTINLL program [54].

### 4.6. Determination of MIC and MBC Values

The MIC and MBC values of the Na14 peptide were determined by broth microdilution against the tested bacteria, including *E. coli* TISTR 887, *P. aeruginosa* TISTR 357, *K. pneumoniae* TISTR 1383, *S. aureus* TISTR 517, and MRSA strain 2468. A bacterial suspension was prepared by adjusting its turbidity at OD 625 nm to 0.1 and subsequently made a 20-fold dilution using Cation-Adjusted Mueller–Hinton broth (CAHMB) as a diluent. One hundred microliters of the Na14 peptide in CAHMB, ranging from 0.25 to 128 µg/mL, was transferred into the 96-well plates. The diluted cell suspension (10 µL) was added to each well, and the plates were incubated at 37 °C for 24 h. The CAMHB alone was used as a negative control, while CAMHB containing colistin or vancomycin served as a positive control. The minimum inhibitory concentration (MIC) was defined as the lowest concentration of the Na14 peptide that inhibits visible bacterial growth. To determine the minimum bactericidal concentration (MBC), 100 µL of the samples from each well were plated onto MH agar and incubated at 37 °C for 24 h. The MBC was identified as the lowest concentration of the Na14 peptide at which no bacterial colonies were observed on the agar plate following incubation [55].

### 4.7. Stability Assessment of the Peptide Under Heat, pH, and Proteolytic Enzyme Conditions

The stability of the peptide was evaluated under various conditions that may influence its antimicrobial activity [30]. The purified Na14 AMP was prepared at a concentration of 10× MIC. Thermal stability was assessed by incubating the peptide at 30 °C, 50 °C, 70 °C, and 90 °C for 3 h. pH stability was evaluated by adjusting the peptide solution to pH 3, 5, 7, 9, and 11 using 1 N HCl or 1 N NaOH, followed by incubation at 37 °C for 3 h and subsequent neutralization to the original pH. To assess enzymatic stability, the peptide was incubated with trypsin, α-chymotrypsin, and proteinase K at a final concentration of 1 mg/mL (Sigma-Aldrich, Warren, MI, USA) at 37 °C for 3 h. The residual antimicrobial activity of each treatment condition was determined using an agar well diffusion assay against *E. coli* TISTR 887, *P. aeruginosa* TISTR 357, and *K. pneumoniae* TISTR 1383 to cross-validate efficacy across multiple pathogens. The untreated peptide served as a control. Statistical significance was analyzed using one-way ANOVA, with comparisons to the control group considered significant at *p*-value < 0.05. The results are presented as the percentage of residual antimicrobial activity.

### 4.8. Time–Kill Assay

Bacterial suspensions of *E. coli* TISTR 887, *P. aeruginosa* TISTR 357, and *K. pneumoniae* TISTR 1383 were prepared by adjusting their turbidity to an OD of 0.1 at 625 nm. The cultures were diluted 20-fold using CAMHB. Subsequently, 100 µL of the Na14 peptides in CAMHB, at concentrations corresponding to 0.5×, 1×, and 2× MIC, were dispensed into the 96-well microplates. The diluted bacterial suspension (10 µL) was mixed into each well before incubating the plates at 37 °C. At predetermined time points, 100 µL of either diluted or undiluted samples from each well were plated on MH agar and incubated at 37 °C for 24 h. Bacterial viability was assessed by colony counting and expressed as log CFU/mL [56].

### 4.9. Morphological Assessment of Bacterial Cells Treated with AMP Using SEM

The morphological effects of the Na14 peptide on *E. coli* TISTR 887, *P. aeruginosa* TISTR 357, and *K. pneumoniae* TISTR 1383 were evaluated using SEM. Overnight cultures (18 h) grown in MH broth were harvested by centrifugation and rinsed twice with sterile 0.9% NaCl. Cell suspensions were then adjusted in CAMHB to an OD of 0.1 at 625 nm. Aliquots were incubated with the AMP at 1× MIC for 16 h. For SEM preparation, treated and control cells were fixed in 2.5% glutaraldehyde in 0.1 M phosphate buffer (pH 7.2) for 24 h, followed by dehydration through a graded ethanol series ranging from 0% to 100%. Residual ethanol was finally removed using a critical point drying machine (Quorum Technologies Ltd., Lewes, East Sussex, UK), after which the specimens were coated with a thin layer of gold. Images were captured at 20,000× magnification [48]. Bacterial morphologies following AMP exposure were compared with those of cells treated with colistin at 1× MIC.

### 4.10. Genomic Analysis and Taxonomic Classification of the Na14 Strain

A single colony of Na14 strain was cultured on MH agar for 24 h before phenotypic and genotypic identification for taxonomic analysis. Gram staining and endospore-staining were performed, and cells were observed under a compound light microscope at 1000× magnification to determine Gram reaction and endospore formation (Carl Zeiss, Oberkochen, Germany) [57,58]. Genomic DNA was extracted from the bacterial culture and sequenced using the Illumina HiSeq platform with 150 bp paired-end reads (Illumina, San Diego, CA, USA), provided by U2Bio Co., Ltd. (Seoul, Republic of Korea). Raw paired-end sequencing reads underwent quality control and genome assembly using the Galaxy Australia platform (version 23.1) [59]. The quality of raw reads was assessed using FastQC (version 0.12.1) both before and after adapter trimming. Reads shorter than 30 base pairs were filtered out, and adapter sequences were removed using Fastp (version 0.23.4) [60]. *De novo* genome assembly was performed using the SPAdes genome assembler (version 3.14.1) via the Shovill platform (version 1.1.0). The quality of the assembled genome and its completeness were subsequently assessed using QUAST (version 5.2.0) [61] and CheckM (version 1.0.18), respectively [62]. Taxonomic analysis and genome comparison of the Na14 strain were performed using the Type (Strain) Genome Server (TYGS). Species-level identification was conducted based on digital DNA−DNA hybridization (dDDH) using the Genome BLAST Distance Phylogeny (GBDP) method [63]. The GBDP results were used to construct the phylogenetic tree using MEGA X software (version 10.1.8) [64]. The identified genotypic information of the Na14 strain was deposited in the GenBank database, accompanied by gene annotation performed using the Prokaryotic Genome Annotation Pipeline (PGAP, version 6.10) via the NCBI platform [65,66,67]. The secondary metabolism and antimicrobial peptide production potential of the Na14 strain were assessed through the prediction of biosynthetic gene clusters (BGCs) based on genomic data using the antiSMASH platform (version 7.0) [68]. Antimicrobial resistance genes were predicted using genomic data through the Resistance Gene Identifier (RGI) tool available on the Comprehensive Antibiotic Resistance Database (CARD) [69].

### 4.11. Comparative Structural Analysis of NRPS Modules from Genomic Data

Insight into the enzymes involved in AMP production was obtained through bacterial genome analysis and comparative approaches using various bioinformatic tools. The amino acid sequences encoded by the NRPS modules, predicted by antiSMASH, were modeled using the AlphaFold Server (https://alphafoldserver.com, accessed on 20 March 2025) [70]. The amino acid sequences and predicted protein structures were compared with reference protein structures deposited in the PDB database to assess structural similarity. The comparison was performed using the FoldSeek server, which identified the best-matched reference proteins based on ranking scores, including root-mean-square deviation (RMSD), template modeling score (TM-score), and sequence identity [71]. The structural comparison of each domain within the predicted NRPS modules was visualized using Visual Molecular Dynamics (VMD, version 1.9.3) and presented by overlaying the predicted protein structures with their corresponding reference structures [72]. The proposed functions of the predicted NRPS modules were assigned based on the annotated functions of reference proteins in the Pfam database, accessed through links provided by the PDB database [73].

## 5. Conclusions

In response to the urgent need for new therapeutics against antimicrobial-resistant Gram-negative pathogens, this study identified *Paenibacillus* sp. Na14 as a novel source of a potent AMP. The Na14 peptide displayed typical AMP characteristics, including positive charge and hydrophobicity. It exhibited a rapid killing rate and strong bactericidal activity against Gram-negative bacteria. This peptide demonstrated comparable effectiveness to colistin. Its mode of action was confirmed to involve membrane disruption. Genomic analysis revealed BGCs associated with the NRPS system, providing valuable insights into the AMP biosynthesis pathway. These findings not only expand the current understanding of naturally derived AMPs but also highlight the Na14 peptide as a promising drug candidate for the development of novel therapies targeting multidrug-resistant Gram-negative infections. To advance this discovery toward clinical application, future studies should include chemical synthesis of the peptide to confirm its activity and enable structure–activity relationship (SAR) analysis. Comprehensive *in vivo* efficacy, toxicity, and safety assessments, followed by clinical evaluation, will be essential to support its therapeutic potential. Furthermore, molecular genetic studies are needed to experimentally validate the predicted AMP biosynthetic enzymes, facilitating the rational development of Na14-derived drug candidates.

## Figures and Tables

**Table 1:** Antimicrobial activity of the CFS derived from the Na14 isolate. The diameter of the inhibition zones observed in the agar well diffusion assay was measured.

Sample	Zone of Inhibition (Mean ± SD; *n* = 3)				
	*E. coli* TISTR 887 (mm)	*P. aeruginosa* TISTR 357 (mm)	*K. pneumoniae* TISTR 1383 (mm)	*S. aureus* TISTR 517 (mm)	MRSA Strain 2468 (mm)
CFS of the Na14 isolate	18.44 ± 0.26	18.34 ± 0.49	18.19 ± 0.46	11.33 ± 0.41	12.45 ± 0.73
Colistin (25 μg)	26.52 ± 0.43	29.41 ± 0.65	26.67 ± 0.22	0.00 ± 0.00	0.00 ± 0.00
Vancomycin (30 μg)	0.00 ± 0.00	0.00 ± 0.00	0.00 ± 0.00	28.50 ± 0.22	28.96 ± 0.43

**Table 2:** Purification balance sheet of the Na14 peptide against *E. coli* TISTR 887.

Purification Step	Volume (mL)	Total Weight (mg)	Arbitrary Activity (AU/mL)	Total Activity (AU)	Specific Activity (AU/mg)	Purification Fold	Yield (%)
Cell-free supernatant	1000	2140.12	40	40,000	18.69	1.00	100.00
Salt precipitation	80	212.23	160	12,800	60.31	3.23	32.00
CIEX	55	87.45	160	8800	100.63	5.38	22.00
RPC	18	6.89	320	5760	835.99	44.73	14.40

**Table 3:** Predicted secondary structure of the Na14 peptide. The spectra of the purified Na14 peptides in deionized water or 50 mM SDS solution were determined by CD. The CONTINLL method was used to predict the peptide secondary structures.

Solvents	α-Helix	β-Strand	Turn	Random Coil
deionized H_2_O	1.7	17.8	23.7	56.8
50 mM SDS	24.4	16.0	15.7	43.9

**Table 4:** The MIC and MBC values of the Na14 peptide against the tested bacterial strains were determined using the broth microdilution assay. The standard antibiotics, including colistin and vancomycin, were used as controls. All experiments were conducted in triplicate.

**Substance**	**Gram-Negative Bacteria**					
	***E. coli* TISTR 887**		***P. aeruginosa* TISTR 357**		***K. pneumoniae* TISTR 1383**	
	**MIC** (**µg/mL**)	**MBC** (**µg/mL**)	**MIC** (**µg/mL**)	**MBC** (**µg/mL**)	**MIC** (**µg/mL**)	**MBC** (**µg/mL**)
Na14 peptide	2	2	2	2	4	4
Colistin	1	1	2	2	1	1
**Substance**	**Gram-Positive Bacteria**					
	***S. aureus* TISTR 517**		**MRSA strain 2468**			
	**MIC** (**µg/mL**)	**MBC** (**µg/mL**)	**MIC** (**µg/mL**)	**MBC** (**µg/mL**)		
Na14 peptide	>128	>128	>128	>128		
Vancomycin	2	2	4	4		

**Table 5:** The residual antimicrobial activity of the Na14 peptide against the tested bacterial strains was evaluated following exposure to various conditions. Results are expressed as the mean ± standard deviation (SD) from three independent experiments.

Treatment Condition	Residual Antimicrobial Activity of Na14 Peptide (%)		
	*E. coli* TISTR 887	*P. aeruginosa* TISTR 357	*K. pneumoniae* TISTR 1383
	**Temperature treatment**		
Untreated sample	100.00 ± 1.73	100.00 ± 0.37	100.00 ± 2.54
30 °C	98.24 ± 1.67	98.08 ± 1.11	100.89 ± 0.39
50 °C	99.12 ± 1.02	98.51 ± 0.37	98.21 ± 4.09
70 °C	98.89 ± 2.03	98.72 ± 0.64	98.66 ± 4.07
90 °C	98.46 ± 1.55	97.22 ± 1.62	99.11 ± 1.39
	**pH treatment**		
Untreated sample	100.00 ± 0.98	100.00 ± 2.78	100.00 ± 2.08
pH 3	100.64 ± 2.30	99.58 ± 0.74	100.43 ± 0.37
pH 5	100.85 ± 1.95	99.36 ± 1.69	99.14 ± 1.35
pH 7	99.36 ± 2.93	99.36 ± 3.18	98.06 ± 2.34
pH 9	98.72 ± 1.28	98.73 ± 2.30	97.62 ± 2.08
pH 11	98.51 ± 1.33	98.30 ± 2.24	99.57 ± 2.08
	**Enzymatic treatment**		
Untreated sample	100.00 ± 1.89	100.00 ± 2.26	100.00 ± 1.14
Trypsin	89.25 ± 0.43 *	88.72 ± 0.75 *	89.53 ± 1.30 *
α-Chymotrypsin	96.50 ± 1.56 *	93.23 ± 2.60 *	94.01 ± 1.50 *
Proteinase K	96.25 ± 1.30 *	94.24 ± 1.57 *	95.51 ± 0.75 *

* significance at *p*-value < 0.05 compared to its corresponding untreated sample.

**Table 6:** Structure prediction and structural similarity analysis of the NRPS from the Na14 genome compared to reference protein structures in the PDB.

Similarity of the Predicted NRPS Module of Na14 to Reference Proteins	Backbone Overlay of Protein Structure (Pink = Reference Protein, Green = Na14’s Predicted Protein)	Surface Overlay of Protein Structure (Pink = Reference protein, Green = Na14’s Predicted Protein)
**Reference Protein** **PDB ID:** 5ES8 Linear gramicidin synthetase subunit A (thiolation state) **Organism:** *Brevibacillus parabrevis* **Seq. identity:** 39.5% **TM-score:** 0.83386 **RMSD:** 6.23	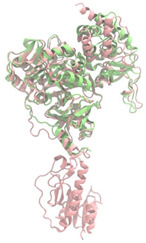	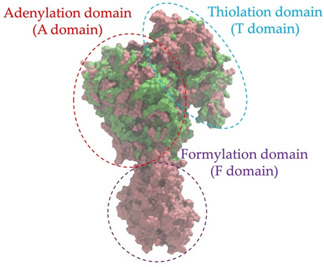
**Reference Protein** **PDB ID:** 6MFZ Linear gramicidin synthetase subunit A (condensation state) **Organism:** *Brevibacillus parabrevis* **Seq. identity:** 31.9% **TM-score:** 0.75324 **RMSD:** 14.72	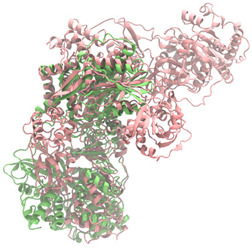	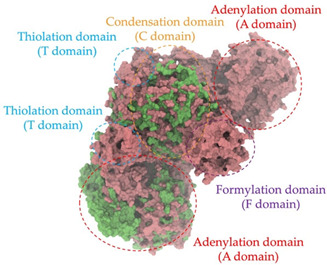
**Reference Protein** **PDB ID:** 2VSQ Surfactin A synthetase C termination module **Organism:** *Bacillus subtilis* **Seq. identity:** 36.6% **TM-score:** 0.70210 **RMSD:** 14.25	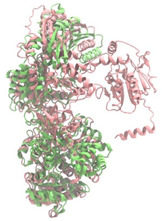	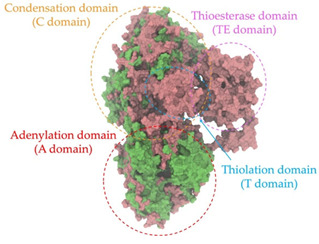
**Reference Protein** **PDB ID:** 6MFZ Linear gramicidin synthetase subunit A (condensation state) **Organism:** *Brevibacillus parabrevis* **Seq. identity:** 38.7% **TM-score:** 0.74215 **RMSD:** 14.05	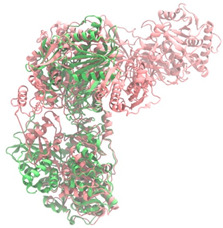	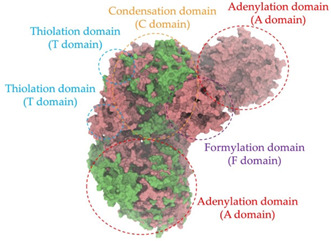
**Reference Protein** **PDB ID:** 2VSQ Surfactin A synthetase C termination module **Organism:** *Bacillus subtilis* **Seq. identity:** 36.1% **TM-score:** 0.70370 **RMSD:** 14.52	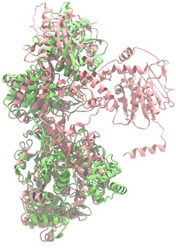	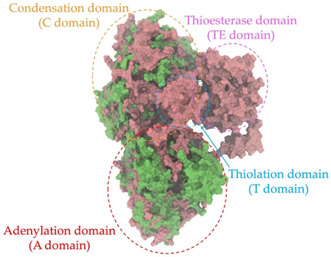
**Reference Protein** **PDB ID:** 2VSQ Surfactin A synthetase C termination module **Organism:** *Bacillus subtilis* **Seq. identity:** 36.4% **TM-score:** 0.71682 **RMSD:** 13.99	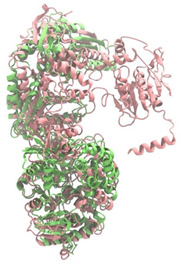	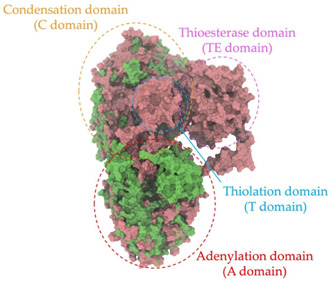
**Reference Protein** **PDB ID:** 2VSQ Surfactin A synthetase C termination module **Organism:** *Bacillus subtilis* **Seq. identity:** 36.8% **TM-score:** 0.77661 **RMSD:** 7.74	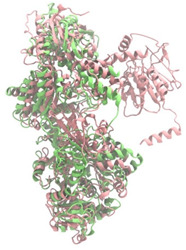	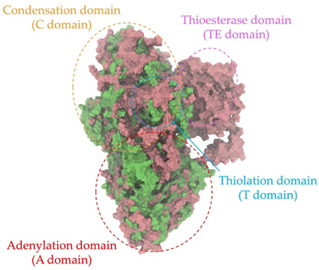
**Reference Protein** **PDB ID:** 6P1J Teixobactin-producing nonribosomal peptide synthetase Txo2 serine module **Organism:** *Eleftheria terrae* **Seq. identity:** 27.0% **TM-score:** 0.77945 **RMSD:** 10.72	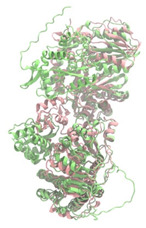	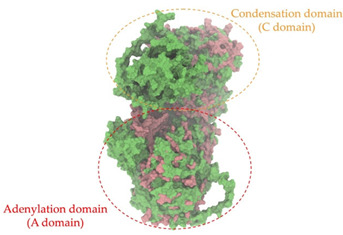
**Reference Protein** **PDB ID:** 2VSQ Surfactin A synthetase C termination module **Organism:** *Bacillus subtilis* **Seq. identity:** 34.0% **TM-score:** 0.46144 **RMSD:** 30.72	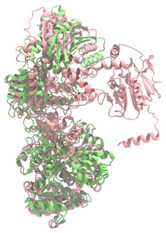	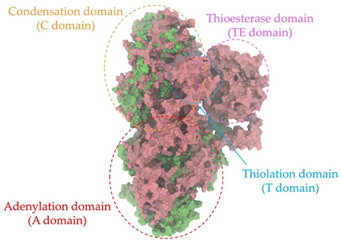
**Reference Protein** **PDB ID:** 6MFZ Linear gramicidin synthetase subunit A (condensation state) **Organism:** *Brevibacillus parabrevis* **Seq. identity:** 40.2% **TM-score:** 0.78563 **RMSD:** 14.37	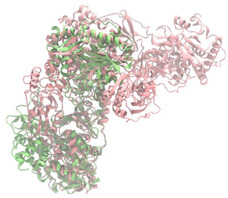	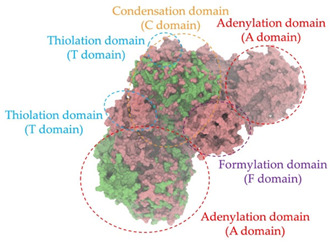
**Reference Protein** **PDB ID:** 2VSQ Surfactin A synthetase C termination module **Organism:** *Bacillus subtilis* **Seq. identity:** 41.7% **TM-score:** 0.66952 **RMSD:** 15.41	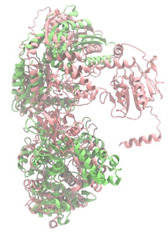	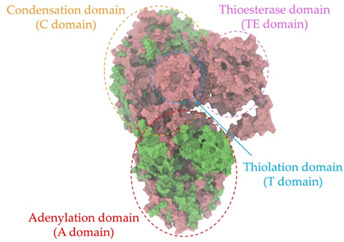
**Reference Protein** **PDB ID:** 6MFZ Linear gramicidin synthetase subunit A (condensation state) **Organism:** *Brevibacillus parabrevis * **Seq. identity:** 36.2% **TM-score:** 0.88822 **RMSD:** 4.01	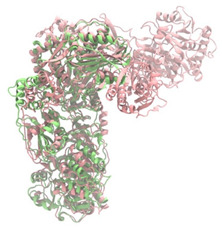	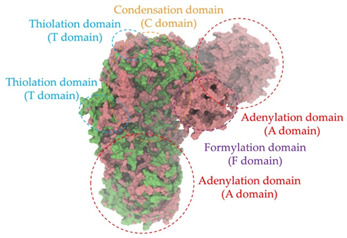
**Reference Protein** **PDB ID:** 6MFZ Linear gramicidin synthetase subunit A (condensation state) **Organism:** *Brevibacillus parabrevis * **Seq. identity:** 38.4% **TM-score:** 0.76129 **RMSD:** 16.26	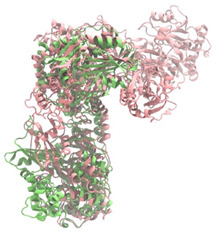	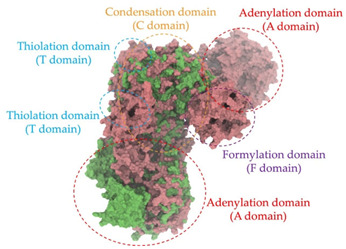
**Reference Protein** **PDB ID:** 2VSQ Surfactin A synthetase C termination module **Organism:** *Bacillus subtilis* **Seq. identity:** 33.7% **TM-score:** 0.63631 **RMSD:** 13.58	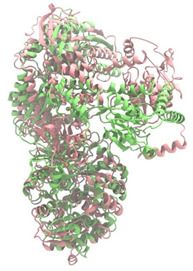	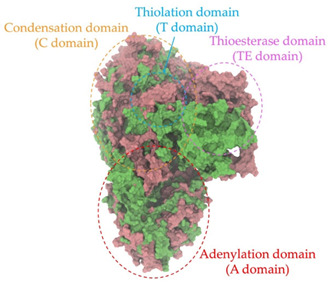

## Data Availability

Data are available within the article. Reasonable inquiries for additional information can be directed to the corresponding author.

## Supplementary Materials

The following supporting information can be downloaded at https://www.mdpi.com/article/10.3390/antibiotics14080805/s1: Figure S1: Preliminary screening of the antimicrobial activity of the Na14 strain using cross-streak method; Figure S2: Antimicrobial activity of the CFS from the Na14 strain using agar well diffusion assay.
