# Supplementary material for: Discovery of a Novel Antimicrobial Peptide from Paenibacillus sp. Na14 with Potent Activity Against Gram-Negative Bacteria and Genomic Insights into Its Biosynthetic Pathway
Source: Antibiotics (Basel). 2025 Aug 6;14(8):805. doi: 10.3390/antibiotics14080805 (PMC12383022; doi:10.3390/antibiotics14080805)
Supplement: Supplementary file 1 [file antibiotics-14-00805-s001.zip › antibiotics-3786277-supplementary.pdf]

Discovery of a Novel Antimicrobial Peptide from *Paenibacillus* sp. Na14 with Potent Activity against Gram-Negative Bacteria and Genomic Insights into Its Biosynthetic Pathway

Nuttapon Songnaka [1,2], Adisorn Ratanaphan [3], Namfa Sermkaew [1,2], Somchai Sawatdee [1,2], Sucheewin Krobthong [4], Chanat Aonbangkhen [4,5], Yodying Yingchutrakul [6] and Apichart Atipairin [1,2,*]

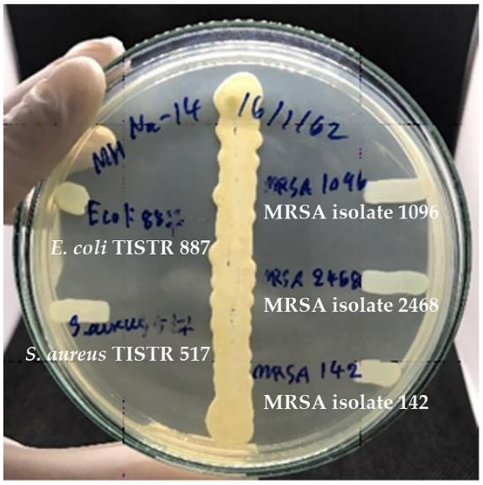

Figure S1. Preliminary screening of the antimicrobial activity of the Na14 strain using cross-streak method. The Na14 strain was streaked onto MH agar and incubated at 37 °C for 4 d. The tested pathogens were streaked perpendicularly to the Na14 streak. The plates were then incubated at 37 °C for 24 h before the zones of inhibition were measured.

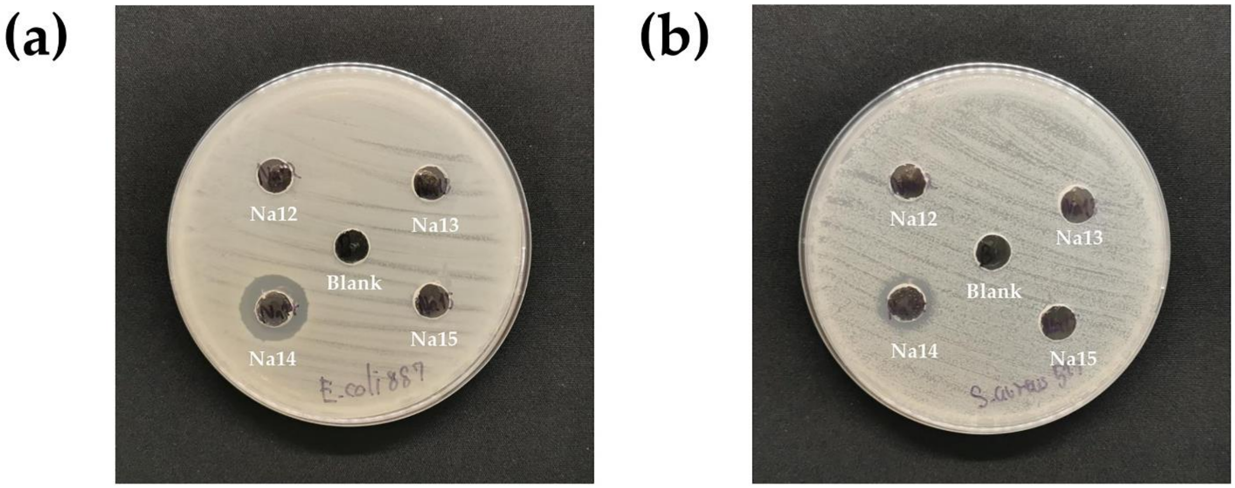

Figure S2. Preliminary screening of the antimicrobial activity of the Na14 strain using agar well diffusion assay. One hundred microliters of the CFS from each isolated strain (Na12, Na13, Na14, and Na15) were loaded into wells on MH agar plates previously precultured with either (a) *E. coli* TISTR 887 or (b) *S. aureus* TISTR 517. The plates were then incubated at 37 °C for 24 h before the zones of inhibition were measured.
